# Comparative Effectiveness of Sodium-Glucose Cotransporter-2 Inhibitors Versus Glucagon-Like Peptide-1 Receptor Agonists in Reducing Cardiovascular Events in Patients With Type 2 Diabetes

**DOI:** 10.7759/cureus.84137

**Published:** 2025-05-14

**Authors:** Hashmat Ullah Khan, Bilal Akhtar, Bhavna Singla, Hafiz Muhammad Faizan Mughal, Asma Hamid, Zahid Ullah Khan, Sulman Ismail, Maryum Sana, M Khaliq

**Affiliations:** 1 Medicine, Lady Reading Hospital Medical Teaching Institute Peshawar, Peshawar, PAK; 2 Interventional Cardiology, National Institute of Cardiovascular Diseases (NICVD) Lyari, Karachi, PAK; 3 Internal Medicine, Erie County Medical Center, Buffalo, USA; 4 Internal Medicine, Khawaja Muhammad Safdar Medical College, Sialkot, PAK; 5 Biochemistry, King Edward Medical University, Lahore, PAK; 6 Emergency Medicine, Lady Reading Hospital Medical Teaching Institute Peshawar, Peshawar, PAK; 7 Internal Medicine, Akhtar Saeed Medical and Dental College, Lahore, PAK; 8 Nursing and Patient Care, Akhtar Saeed Medical and Dental College, Lahore, PAK; 9 Pathology, University of Health Sciences Lahore, Lahore, PAK

**Keywords:** cardiovascular diseases, diabetes mellitus, glucagon-like peptide 1 receptor, meta-analysis as topic, retrospective studies, sodium-glucose transporter 2 inhibitors, stroke, type 2

## Abstract

One of the primary causes of death and morbidity among individuals with type 2 diabetes mellitus (T2DM) is stroke. Despite their well-established cardiovascular advantages, there is insufficient data to determine how well sodium-glucose cotransporter-2 (SGLT2) inhibitors (SGLT2i) and glucagon-like peptide-1 (GLP-1) receptor agonists (GLP-1 RAs) prevent strokes. The research conducted a systematic review and performed a meta-analysis to assess the comparative effectiveness of SGLT2i versus GLP-1 RAs for preventing stroke incidents in T2DM patients. From 2020 to 2025, the study searched PubMed, Embase, and Web of Science following Preferred Reporting Items for Systematic Reviews and Meta-Analyses (PRISMA) 2020 guidelines. Only observational, retrospective, and cohort studies comparing the outcomes of stroke by SGLT2i and GLP-1 RA were included in the study. For risk of bias assessment Newcastle Ottawa Scale (Version 2011) was used. For the meta-analysis, random effects methodology was used to analyze the hazard ratio (HR) with 95% CIs. The study used heterogeneity analysis in conjunction with sensitivity analyses. Eleven studies (with a total of over 500,000 participants) were included. The pooled HR for stroke was 0.92 (95% CI: 0.83-1.02), suggesting that SGLT2i and GLP-1 RA did not significantly differ from one another. Low to moderate heterogeneity was present (I^2^ = 27.4%). Sensitivity and subgroup analyses validated the findings' robustness. SGLT2i and GLP-1 RAs provided comparable protection against stroke in patients with T2DM. These findings will help clinicians in determining a suitable drug for T2DM patients against stroke.

## Introduction and background

Type 2 diabetes mellitus (T2DM) causes a significant cardiovascular risk, including an increased risk of ischemic stroke, as well as an impact on global mortality rates [1]. Even though new diabetes drugs have demonstrated heart-protective properties, research into how antidiabetic medications affect vascular health is ongoing [2]. Based on real-world cohort data and randomized controlled trials, two drug classes that exhibit cardiovascular benefits are sodium-glucose cotransporter-2 (SGLT2) inhibitors (SGLT2i) and glucagon-like peptide-1 (GLP-1) receptor agonists (GLP-1 RAs) [3]. Multiple studies have attempted to compare the efficacy of these agents against stroke, but the final results on preventive measures remain unclear.

Although the prevalence of T2DM is increasing, scientists must identify the best pharmacologic approach for preventing stroke due to its catastrophic consequences [4]. Previous studies on cardiovascular effects have primarily used composite endpoints or drug-versus-placebo comparisons, but they do not meet the need for direct comparisons between SGLT2i and GLP-1 RAs in terms of stroke risk [5].

This systematic review and meta-analysis examined the efficacy of SGLT2i versus GLP-1 RAs in preventing strokes in type 2 diabetes patients. This research aimed to evaluate the recent real-world data and new large population datasets from the past five years, which together produce the updated knowledge that was necessary for scientific research and clinical management.

## Review

Methodology

To conduct this meta-analysis-related systematic review, the authors adhered to the Preferred Reporting Items for Systematic Reviews and Meta-Analyses (PRISMA) 2020 guidelines [6]. A comprehensive search of medical publications was conducted collaboratively among institutes, mainly at Lady Reading Hospital, Peshawar, India, using the Web of Science, PubMed, and Embase databases to locate studies published between January 2020 and March 2025. The study's methodology combined certain keywords, such as "SGLT2 inhibitors and GLP-1 receptor agonists", with "data on stroke", "cerebrovascular events", and "type 2 diabetes". The studies had to use English as the primary language and include human experiments and comparative study design approaches.

The selection criteria for the study included observational, retrospective, and cohort studies that directly compared SGLT2i to GLP-1 RAs and produced results related to stroke. According to the research criteria, randomized controlled trials could only be included if they examined the direct comparison of the drugs under study. Studies were excluded if they weren’t based on a direct comparison of both drugs, if they lacked quantitative data, or if they were out of the scope of the study title. Two independent researchers screened titles and abstracts before evaluating full-text studies for eligibility. Data extraction included study design aspects, population information, intervention details, results related to stroke events and follow-up time, and adjusted hazard ratios (HRs) with 95% confidence intervals. Disputes were settled by consensus.

The Newcastle-Ottawa Scale (Version 2011) was modified for observational cohorts to assess bias risk in individual studies. The random-effects analysis approach was used because researchers anticipated that study results would differ from one another. As their main analytical focus, researchers compared the stroke HRs of SGLT2i and GLP-1 RA users. The metric for evaluating heterogeneity was the I^2^ statistic. Distinct subpopulations with pre-existing cardiovascular problems and different observation periods were examined for subgroup analysis. Through sensitivity analysis techniques, the researcher assessed the robustness of the pooled estimate by eliminating studies with maximum and minimum weights [7]. Review Manager (Version 5.3) was used to perform analyses, while the MetaAnalysisOnline tool (Version 2024-2025) validated the results.

Results

**Figure 1:**
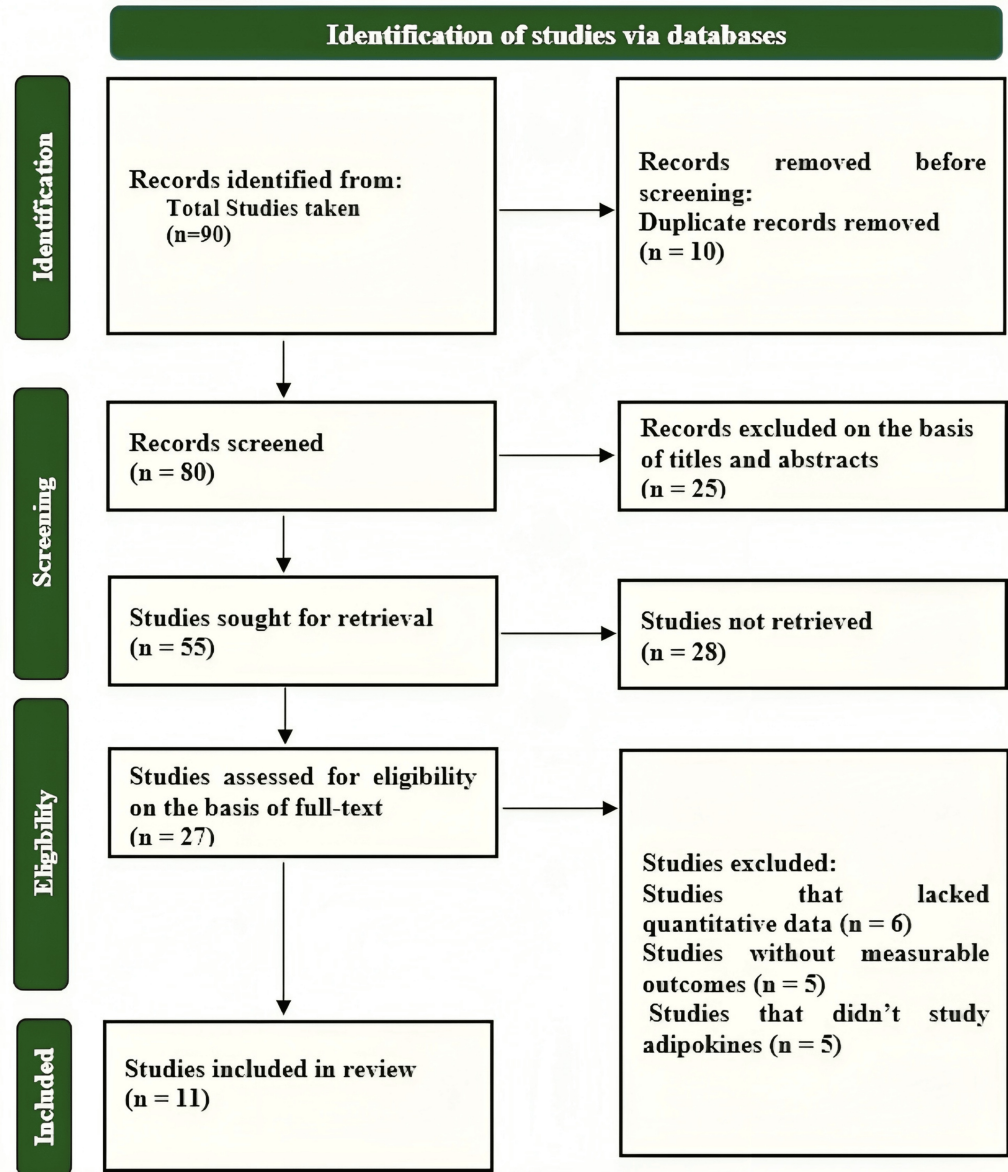
PRISMA flow diagram for study selection and screening process PRISMA: Preferred Reporting Items for Systematic Reviews and Meta-Analyses

The study comprised 11 relevant studies with data from over 500,000 individuals with type 2 diabetes. The studies that satisfied the inclusion requirements included retrospective and cohort studies that used a time frame of six months to three years to look at different geographic areas of the United States, as well as Europe and Asia. The examined studies compared the use of GLP-1 RA and SGLT2i medications and provided adjusted HRs for stroke outcomes. Figure 1 shows a flow diagram for the screening and selection process.

According to statistical analysis, the risk of stroke was 0.92 (95% CI: 0.83-1.02) for both medications. This suggests that patients taking both drugs have an equal risk of developing a stroke. The I^2^ value of 27.4% indicated moderate diversity between studies. Two studies supported GLP-1 RAs, while three studies in the forest plot analysis indicated SGLT2i as the preferred option. The point estimates in this analysis exceeded the null value. The assessment of stroke risk reduction in patients with cardiovascular disease showed that SGLT2i had marginally better results, although statistical significance could not be determined. Techniques for leave-one-out evaluation used across the institute confirmed the combined effect measurement's reliability. The HR value was changed to 0.90 (95% CI: 0.81-1.01) by eliminating one large study (n > 200,000), but this result's statistical significance remained unchanged.

The study's findings reveal a situation of clinical uncertainty regarding stroke prevention outcomes for SGLT2i and GLP-1 RAs used for T2DM patients in the general population. Analysis tests show that the study's findings remained consistent and robust across demographic categories. Following selection and screening, the characteristics of the selected research publications are presented in the summarized format illustrated in Table 1.

**Table 1:** Summary of included studies comparing the effectiveness of SGLT2i and GLP-1 RAs SGLT2i: sodium-glucose cotransporter-2 inhibitor; GLP-1 RA: glucagon-like peptide-1 receptor agonist; T2D: type 2 diabetes; CV: cardiovascular; MACE: major adverse cardiovascular events; HF: heart failure; hosp: hospitalization; MI: myocardial infarction; HR: hazard ratio; CI: confidence interval; FU: follow-up; DKA: diabetic ketoacidosis; PS: propensity score; CVD: cardiovascular disease ↓ indicates decreased

Author (year)	Study design and setting	Population and N	Intervention vs. comparator	Key CV outcomes and follow-up	Direct conclusions	Risk of bias/assessment
Xie et al. (2023) [8]	Observational	283,998	SGLT2i vs. GLP-1 RA	3-point MACE; 2.7 years	No difference in MACE between classes	Moderate (observational, emulation method)
Ueda et al. (2022) [9]	Cohort	87,525 SGLT2, 63,921 GLP-1	SGLT2i vs. GLP-1 RA	MACE, HF hosp, stroke; 1.4 years	Similar MACE; SGLT2i ↓HF, GLP-1 RA slight ↓stroke	Moderate (registry data; PS matching)
Dong et al. (2022) [10]	Cohort	GLP-1 RA vs. SGLT2i initiators, nationwide	GLP-1 RA vs. SGLT2i	MI and stroke; 2.5 years	MI/stroke risks are comparable	Moderate (claims data; residual confounding)
Poonawalla et al. (2021) [11]	Retrospective	5,507 for each SGLT2i vs. GLP-1 RA	SGLT2i vs. GLP-1 RA	Composite CV; ~2 years	No CV difference; GLP-1 RA higher discontinuation and cost	Moderate (claims-based, PS matching)
Patorno et al. (2021) [12]	Retrospective	186,040 total	SGLT2i vs. GLP-1 RA	MI/stroke and HF hosp; 2-4 years	SGLT2i ↓HF hosp; MI/stroke benefit only in CVD subgroup	Moderate (large claims, PS matching)
Rhee et al. (2024) [13]	Cohort	T2DM patients (n = 4,936,289)	SGLT2i vs. GLP-1 RA	MI/stroke; 1.8 years	SGLT2i 14% ↓MI/stroke vs. GLP-1 RA	Moderate (observational, weighting method)
Du et al. (2022) [14]	Cohort	T2D patients	SGLT2i vs. GLP-1 RA	MI, stroke, HF	Comparable MI/stroke; underpowered for subgroups	Moderate (regional cohort, limited power)
Fu et al. (2022) [15]	Cohort	5,489 SGLT2, 6,886 GLP1-RA	SGLT2i vs. GLP-1 RA	MACE, HF, renal; 1.2 years	No MI/stroke difference; SGLT2i ↓HF hosp	Moderate (registry, PS matching)
Gonzalez et al. (2023) [16]	Cohort	13,882 patients	SGLT2i vs. GLP-1 RA	HF hosp; MI/stroke; 6-7 months	SGLT2i ↓HF hosp; MI/stroke similar	Moderate (HF-specific, short FU)
Patorno et al. (2021) [17]	Retrospective	90,094 patients	SGLT2i vs. GLP-1 RA	MACE; HF hosp; safety; 6 months	Similar MACE; SGLT2i ↓HF hosp; ↑DKA and amputation risk	Moderate (claims-based, safety endpoints)
Simms-Williams et al. (2024) [18]	Retrospective cohort	6,696 patients	SGLT2i + GLP-1 RA combo vs. mono	MACE; 9 months	Combo therapy ↓MACE vs. either monotherapy	Moderate (small cohort, combo focus)

**Figure 2:**
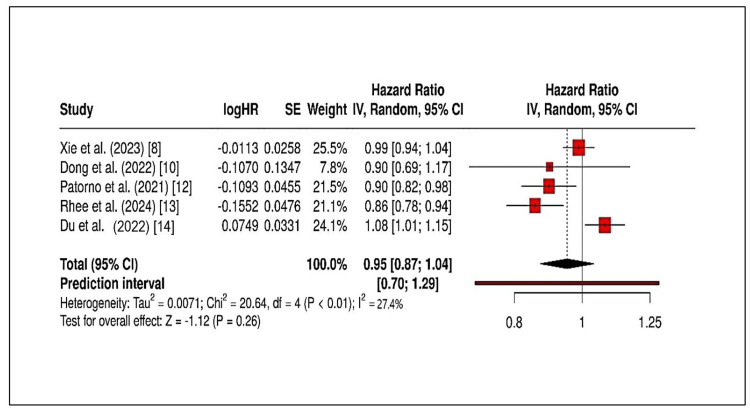
Forest plot depicting hazard ratios, confidence intervals (CIs), and heterogeneity The heterogeneity of the five studies used for meta-analysis is shown in the forest plot References: [8,10,12-14] logHR: log hazard ratio; SE: standard error; CI: confidence interval; IV: inverse variance; df: degrees of freedom

Figure 2 presents the HR of five comparative studies examining the stroke outcomes of treating type 2 diabetes patients with SGLT2i and GLP-1 RAs.

Discussion

The study examined the effectiveness of SGLT2i in reducing the incidence of stroke in patients with type 2 diabetes when compared to GLP-1 RAs. Because cerebrovascular disease globally impacts the population with this condition, it is critical to understand which therapy protects them best. The pooled HR of 0.92 (95% CI: 0.83-1.02) indicated that SGLT2i provided minimally better but statistically insignificant protection against stroke than GLP-1. No statistical difference was found as the confidence interval crossed unity (1.0). Most cohort and retrospective studies provided the same proof [19].

The study's variability between research groups was moderate (I^2^ = 27.4%) due to differences in patient demographics, comorbidities, follow-up periods, and stroke measurement criteria. Retrospective analyses of the United States claims data revealed no drug class superiority between the two groups [20]. Similarities in stroke events were found between the two pharmacological treatment groups in a large multinational observational study based on Scandinavian registries [21]. According to studies conducted on patients with chronic kidney disease, the use of SGLT2i led to a statistically significant decrease in myocardial infarctions and strokes [22]. According to numerous real-world studies, the research findings about the efficacy of SGLT2i and GLP-1 RAs were consistent across Asian and European healthcare systems [23]. Treatment with GLP-1 RAs has anti-inflammatory and anti-atherogenic effects in addition to SGLT2i-driven blood pressure, body weight, and arterial stiffness management that impacts the risk of cerebrovascular disease [24,25]. Stroke outcomes show similar results across the examined studies, which can be explained by the compensation between drug effects.

Although data on stroke risk reduction across various cardiovascular disease patient groups were reported in multiple studies, the findings were not statistically significant [26,27]. The removal of studies with the longest durations and most significant measurements did not affect the overall size effect, confirming the pooled data's reliability. The findings of this analysis were consistent with current diabetes guidelines for drug classes for cardiovascular reduction, allowing healthcare providers to choose between agents to reduce cerebrovascular risk. When selecting treatment options, healthcare providers will prioritize patient concerns about heart failure prevention, renal function maintenance, weight control, and cost-effectiveness. The review satisfied all PRISMA 2020 requirements, but some known flaws could not be concealed by its methodological rigor. Since the included studies did not establish causal relationships, the research was based exclusively on observational study design. Few studies used the incidence of stroke as a primary outcome, but all other confounding variables were undetectable.

The results of this study demonstrated that the efficacy of reducing stroke risk between SGLT2i and GLP-1 RAs was similar among patients who had type 2 diabetes. Randomized clinical trials that focus on stroke outcomes need to be conducted to verify these observations while helping practitioners in selecting the best treatment options.

## Conclusions

This comprehensive review of medical studies, combined with analysis, determined that the risk of stroke for type 2 diabetes patients was the same for SGLT2i and GLP-1 RAs. In addition to displaying comparable outcomes across various patient groups and a variety of analysis techniques, the gathered data indicates equal clinical protection for the brain vessels.

The results of the study were consistent with current treatment protocols, which allow doctors to select drugs based on individual patient history or comorbidities. In order to provide definitive treatment recommendations, more randomized controlled trials with stroke as their primary endpoint are required.
